# Role of Race/Ethnicity, Language, and Insurance in Use of Cervical Cancer Prevention Services Among Low-Income Hispanic Women, 2009–2013

**DOI:** 10.5888/pcd15.170267

**Published:** 2018-02-22

**Authors:** John Heintzman, Brigit Hatch, Gloria Coronado, David Ezekiel, Stuart Cowburn, Octavio Escamilla-Sanchez, Miguel Marino

**Affiliations:** 1 Oregon Health and Science University, Portland, Oregon; 2 OCHIN, Inc, Portland, Oregon; 3 Kaiser Permanente Center for Health Research, Portland, Oregon; 4 Antioch College, Yellow Springs, Ohio

## Abstract

**Introduction:**

Hispanic women in the United States have an elevated risk of cervical cancer, but the existing literature does not reveal why this disparity persists.

**Methods:**

We performed a retrospective cohort analysis of 17,828 low-income women aged 21 to 64 years seeking care at Oregon community health centers served by a hosted, linked electronic health record during 2009 through 2013. We assessed the odds of having had Papanicolaou (Pap) tests and receiving human papillomavirus (HPV) vaccine, by race/ethnicity, insurance status, and language.

**Results:**

Hispanic women, regardless of pregnancy status or insurance, had greater odds of having had Pap tests than non-Hispanic white women during the study period. English-preferring Hispanic women had higher odds of having had Pap tests than Spanish-preferring Hispanic women (OR, 2.08; 95% confidence interval [CI], 1.63–2.66) but lower odds of having received HPV vaccination (OR, 0.21; 95% CI, 0.12–0.38). Uninsured patients, regardless of race/ethnicity, had lower odds of HPV vaccine initiation than insured patients did. Once a single dose was received, there were no significant racial/ethnic differences in vaccine series completion.

**Conclusion:**

In this sample of low-income women seeking care at Oregon community health centers, we found minimal racial/ethnic disparities in the receipt of cervical cancer prevention services. Inequities by insurance status, especially in the receipt of HPV vaccine, persist. Community health center–based care may be a useful model to address racial/ethnic disparities in prevention, but this model would need further population-wide study.

## Introduction

Hispanic women in the United States have an increased risk of cervical cancer (1,2) despite the existence of screening techniques that have longstanding, demonstrated effectiveness (3) and a vaccine that protects against the primary cause of cervical cancer — infection with certain subtypes of human papillomavirus (HPV) (4). Regular Papanicolaou (Pap) testing of women aged 21 to 65 years detects early cervical changes associated with HPV; treatment prevents progression to invasive cervical cancer (3). It is uncertain which biological, social, or behavioral factors might factor most heavily in these cancer prevention disparities. Health services research on the uptake of cervical cancer preventive services (Pap testing and HPV vaccination) among Hispanic women has largely been survey-based and has yielded varying and sometimes contradictory results. Many studies show that Hispanic women are less likely than other women to receive recommended Pap tests (5–7) and suggest numerous factors that may affect this disparate utilization (6,8–12). However, other survey-based studies show that Hispanic women may use Pap tests more than non-Hispanic white women (2,13,14). There is similarly conflicting information about disparities in HPV vaccination uptake; some studies show lower vaccine initiation or completion rates among Hispanic women compared with non-Hispanic white women (15–17), some studies do not show disparities in certain Hispanic subpopulations (15), and some studies suggest impacts of insurance and other factors on the uptake of HPV vaccine (17,18). With some exceptions (17,18), most of this work is survey-based, which has significant limitations in the study of preventive service use (19–22). Survey responses can be influenced by social desirability bias, and, because they are often administered at a single time point, surveys often provide limited information about services and associated factors over time.

We aimed to compare the use of cervical cancer prevention services (ie, Pap testing and HPV vaccination initiation and completion) among Hispanic women and non-Hispanic white women who sought care at community health centers (CHCs) with linked electronic health records (EHRs) during a 5-year period in Oregon. This investigation was novel in its use of objective EHR data, its 5-year study period, and its incorporation of language and insurance status as moderators.

## Methods

This was a retrospective observational cohort study of factors associated with cervical cancer screening and HPV vaccination receipt among female CHC adult patients who had a clinic visit during 2009 through 2013. We used EHR data from OCHIN (not an acronym), a 501(c)(3) network that centrally hosts an Epicare platform to more than 300 CHCs nationwide covering more than 2 million patients (23–25). The Oregon CHCs included in this analysis provide comprehensive primary care to low-income patients in rural, urban, and suburban communities across Oregon, encompassing preventive services and acute, chronic, mental or behavioral, and obstetric care (23). Participants were Hispanic women or non-Hispanic white women aged 21 to 64 years who accessed 1 of the 23 OCHIN-affiliated CHCs in Oregon at least once (26) during 2009 through 2013 and had income of less than 100% of the federal poverty level at every visit (n = 17,828). Women with a documented hysterectomy were excluded from the analysis.

### Variables

Variables were race/ethnicity (Hispanic vs non-Hispanic white), preferred language (Spanish vs English), insurance status (insured with Medicare, Medicaid, and/or private insurance at any point during the study period vs no insurance). We used Hispanic and non-Hispanic white as ethnicity categories, because these reflect the specific federally reported categories collected by CHCs; we acknowledge the limits of these ethnic categories in describing this population.

Outcomes were receipt of a Pap test during the study period, receipt of 1 HPV vaccine dose during the study period, and receipt of a complete 3-dose HPV vaccine series during the study period. We chose these outcomes because they are clinically relevant (1 vaccine dose offers protection against HPV [27]), and because these benchmarks may represent 2 phases of care quality: 1) initial engagement with and 2) completion of a recommended preventive service that takes a period of time. Initial recommendations for HPV vaccine were made by the Advisory Committee on Immunization Practices (ACIP) in 2007 (28), and we were able to capture data on all vaccinations in our network during 2007 through 2013. For some analyses, we restricted the sample to women aged 21 to 29, who, among our overall cohort, might conceivably receive HPV vaccine (even if older than the recommended maximum age of 26). Our study period was largely before widespread implementation of the 2012 recommendation for HPV testing along with cervical cytology evaluation in some populations, so this testing was not included in our outcomes.

Covariates were age at start of the study period, number of CHC visits during the study period (categorized as 1, 2–5, or >5 visits over 5 years), and pregnancy during the study period. CHC use served as a proxy for comorbidity in general (29); we also adjusted for pregnancy, which could have associations with our outcome.

### Statistical analysis

Race/ethnicity and insurance disparities in receipt of cervical cancer screening among the overall sample (N = 17,828) were examined by comparing the odds of receiving at least 1 Pap test and receiving HPV vaccination among 4 cohorts: insured Hispanic women, uninsured Hispanic women, insured non-Hispanic white women, and uninsured non-Hispanic white women. Multivariable logistic regression was used to estimate adjusted odds ratios (aORs) and 95% confidence intervals (CIs). Because patients in the same CHC are more likely to be similar to each other in receipt of services than they are to patients in other CHCs (hence, no longer independent), the Huber–White estimator of the standard error (30) was used to estimate 95% CIs for the adjusted odds ratios to account for clustering of patients within their home clinic (the CHC that the patient visited most often). Secondary analyses examined the associations between cervical cancer prevention services and patients’ preferred language (among Hispanic women only; N = 3,384) and among subsets of women aged 21 to 29 (for HPV vaccine). All secondary analyses also used similar multivariable logistic regression with clustered standard errors similar to the primary analysis. Statistical tests were 2-sided; significance was set at a *P* value less than .05. Statistical analyses were performed in 2016–2017 using R version 3.3.2 (The R Foundation). The institutional review board of Oregon Health and Science University approved this study.

## Results

Of our total study population (N = 17,828), the largest cohort was insured non-Hispanic white women (n = 10,063) (Table). Insured patients (Hispanic women and non-Hispanic white women) were more likely than uninsured patients to be in the youngest (21–29 y) age bracket (43% and 37%, respectively). Most Hispanic women identified Spanish as their preferred language. Both insured cohorts were more likely than the uninsured cohorts to have more than 5 visits during the study period (Hispanic women, 58%; non-Hispanic white women, 49%), and both uninsured cohorts were more likely than the insured cohorts to have 2 to 5 visits during the study period (Hispanic women, 43%; non-Hispanic white women, 44%). Though all cohorts had high percentages of women who were not pregnant during the study period, insured Hispanic women were the largest proportion of pregnant women (47%).

**Table:** Patient Characteristics in Study of Use of Cervical Cancer Prevention Services Among Low-Income Women, by Race/Ethnicity and Insurance Status, Oregon, 2009–2013

Characteristic	N (Column %)					
	Hispanic Women Insured (n = 2,381)	Hispanic Women Uninsured (n = 2,452)	Non-Hispanic White Women Insured (n = 10,063)	Non-Hispanic White Women Uninsured (n = 2,932)	Total (n = 17,828)	*P* Value^a^
**Age, y**						
21–29	1,032 (43)	766 (31)	3,709 (37)	839 (29)	6,346 (36)	<.001
30–39	815 (34)	985 (40)	2,574 (26)	701 (24)	5,075 (28)	
40–49	337 (14)	424 (17)	2,001 (20)	766 (26)	3,528 (20)	
50–64	197 (8)	277 (11)	1,779 (18)	626 (21)	2,879 (16)	
**Language**						
Spanish	1,550 (65)	2,192 (89)	22 (0)	37 (1)	3,801 (21)	<.001
English	804 (34)	246 (10)	9,485 (94)	2,829 (96)	13,364 (75)	
Other	27 (1)	14 (1)	556 (6)	66 (2)	663 (4)	
**Office visits during 2009–2013**						
1	271 (11)	525 (21)	1,803 (18)	1,126 (38)	3,725 (21)	<.001
2–5	726 (30)	1,045 (43)	3,285 (33)	1,286 (44)	6,342 (36)	
>5	1,384 (58)	882 (36)	4,975 (49)	520 (18)	7,761 (44)	
**Pregnant anytime during 2009–2013**						
No	1,271 (53)	2,060 (84)	8,381 (83)	2,816 (96)	14,528 (81)	<.001
Yes	1,110 (47)	392 (16)	1,682 (17)	116 (4)	3,300 (19)	

a *P* values were calculated by using a χ^2^ test.

Overall, 55% of the patients in our cohort received a Pap test during the study period: 65% of Hispanic women and 51% of non-Hispanic white women. After adjusting for age, CHC use, and pregnancy status, insured Hispanic women still had higher odds of receiving a Pap test during the study period compared with insured non-Hispanic white women (OR, 1.35; 95% CI, 1.22–1.50). Uninsured Hispanic women also had higher odds of having received a Pap test during the study period (OR, 1.29; 95% CI, 1.18–1.42) compared with insured non-Hispanic white women. Uninsured non-Hispanic white women had lower odds of receiving a Pap test during the study period than insured non-Hispanic white women (OR, 0.48; 95% CI, 0.44–0.53). Nonpregnant women had similar patterns, odds ratios, and significance as all women.

Overall HPV initiation rates in the subset of women aged 21 to 29 years were approximately 4%, and completion rates were approximately 1.4%. Insured Hispanic women did not differ significantly from insured non-Hispanic white women in their receipt of at least 1 HPV vaccine dose (Figure). Both uninsured groups had lower odds of receiving at least 1 HPV vaccine dose than the insured non-Hispanic white group (Hispanic women: OR, 0.31; 95% CI, 0.17–0.55; and non-Hispanic white women: OR, 0.24; 95% CI, 0.12–0.48). Among women who received at least 1 dose of HPV vaccine, we found no significant difference among the study cohorts in completion of the vaccine series, although the sample size for subpopulation was small (n = 268).

**Figure:**
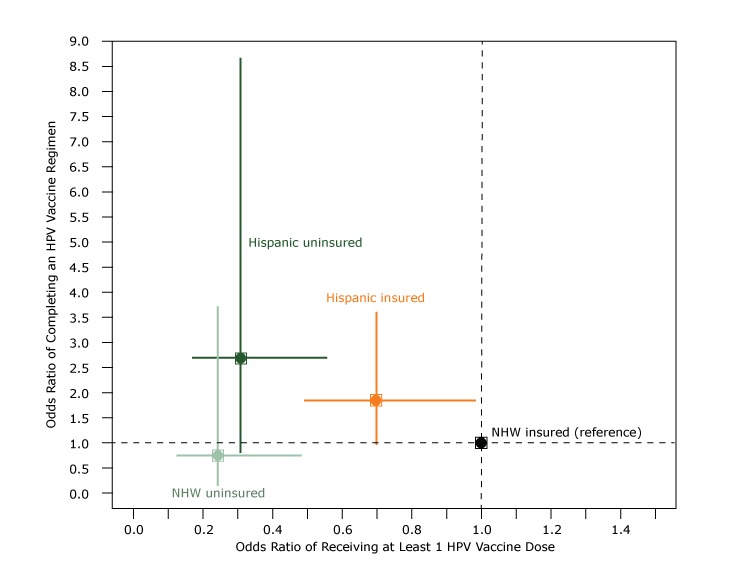
Adjusted odds ratios (ORs) and 95% confidence intervals (CIs) of receiving at least 1 dose of human papillomavirus (HPV) vaccine compared with adjusted odds ratio of completing an HPV vaccine series among women aged 21 to 29 years, by race/ethnicity and insurance status, Oregon, 2009–2013. Multivariable logistic regression was used to estimate odds of receiving at least 1 HPV vaccine dose, by race/ethnicity and insurance status relative to insured non-Hispanic white women (x-axis; n = 6,346). Then, among those who initiated an HPV regimen, we performed another multivariable logistic regression to estimate odds of completing a HPV regimen by race/ethnicity and insurance status relative to insured non-Hispanic white women (y-axis; n = 268). Both models adjusted for pregnancy and number of health center visits (1, 2–5, >5) during the study period. For both models, we estimated 95% confidence intervals using robust Huber–White sandwich estimators of the standard error to account for clustering of patients within home clinic (the community health center that the patient visited most often). Abbreviation: NHW, non-Hispanic white.

In evaluating the impact of preferred language among Hispanic women aged 20 to 29 years, English-preferring Hispanic women had more than double the odds of receiving a Pap test (OR, 2.08; 95% CI, 1.63–2.66) but lower odds of receiving the HPV vaccine (OR, 0.21; 95% CI, 0.12–0.38) than Spanish-preferring Hispanic women (both *P* < .001).

## Discussion

This analysis was a novel investigation of use of cervical cancer preventive services in a large cohort of low-income women in Oregon CHCs. Our findings had several patterns. Overall, the rate of receipt of Pap tests during the 5-year study period (55%) was lower than the national rate of receipt of cervical cancer screening (81%) (31). However, national surveys do not focus on a low-income safety-net population and do not verify self-report with objective confirmation of tests (31), so direct comparison may not be appropriate. In our analysis, Hispanic women (insured and uninsured) had increased odds of receiving a Pap test than insured non-Hispanic white women, even when they were not pregnant. This finding is consistent with some previous research and may suggest that barriers to receiving cervical screening in this population are not directly associated with ethnicity. However, Hispanic women whose language preference was Spanish had a lower screening rate than Hispanic women whose language preference was English. The lower odds of Pap testing among Spanish-speaking Hispanic women was surprising given the language resources available in CHCs and previous findings in our network that showed no difference in other preventive services between English- and Spanish-speaking Hispanics (32,33). This finding needs further research to understand the role of language preference in Pap test uptake.

The HPV vaccination rate among adult women aged 21 to 29 in our network was 4% overall and 3.2% among Hispanic women; these low rates were likely attributable to our inclusion of adults only in the early years of vaccine introduction. Uninsured patients had lower odds of receiving at least 1 HPV vaccine, regardless of whether they were Hispanic women or non-Hispanic white women. Once a single dose was received, there were no observed disparities between cohorts in completion of vaccination, although effect sizes were large for Hispanic women in the direction of Hispanic women having higher odds of completion. This completion of vaccination suggests that lack of insurance is the main barrier to vaccine uptake, a finding that has also been noted in children eligible for this vaccine (17). Spanish language preference was associated with higher odds of receiving vaccine, demonstrating that Spanish preference is not a barrier to the receipt of other vaccines, as other analyses of adult vaccination have noted (32).

Uninsured non-Hispanic white women demonstrated low rates of utilization of services overall. The reasons for these low rates are not certain, but the low rates have been observed in analyses of other services in our network (32–34) and are likely due to additional, unmeasured barriers in this population.

Our study has several limitations. We were unable to definitively capture data on services outside of our network, though evidence suggests patients in our network tend not to receive services elsewhere (35). Our study of HPV vaccine receipt showed low rates overall, limiting the conclusions we can draw from these data and highlighting additional need for research on access to cervical cancer prevention. Our data set did not include adolescents or children, so we could not evaluate HPV vaccination rates among these populations, but because we were able to capture data on all vaccinations from 2007 onward (when the ACIP approved HPV vaccination), we were able to thoroughly evaluate HPV vaccination in this adult cohort. Although we were able to account for insurance, income (our population was low-income), race/ethnicity, and preferred language, there may be social factors (education, employment, marital status) that we were unable to measure but that affect receipt of vaccine. Our approach did not consider a temporal analysis and assumes a negligible temporal trend in Pap testing in these CHCs from 2009 through 2013. This assumption may be feasible because our study period largely predated the widespread use of HPV cotesting (a sentinel policy and practice change). More recent data are needed to replicate our study findings after the introduction of HPV cotesting. Our analysis measures the access to period-appropriate prevention services, not general advances in prevention approach. We were limited to broad federal race/ethnicity categories because more granular categories are not routinely collected in the OCHIN EHR system. An analysis of Hispanic subgroups might yield more nuanced results. Immigration status may also affect use of care, and many patients in our network likely are immigrants (32,34,36). However, we were not able to definitively assess immigration status, so it remains a limitation of our analysis. Finally, our data set did not include data on Pap results, which might have affected receipt of services in meaningful ways and could have been used to study women who develop cervical cancer and the characteristics that put them at higher risk.

In our objective, longitudinal EHR-based analysis, insured and uninsured Hispanic women seen at Oregon CHCs had increased odds of receiving Pap tests compared with insured non-Hispanic white women. Uninsured patients had lower odds of starting the HPV vaccine series, but once initiated, there were no racial/ethnic or insurance disparities in vaccine series completion. Spanish language preference (rather than English preference) among Hispanic women was associated with decreased odds of Pap testing but increased odds of receiving any HPV vaccine. Policy makers should understand that among adult Hispanic women with access to a CHC, cervical cancer prevention services seemed to be equitable, although this observation needs further study in a wider sample of patients. Efforts to ensure that access to cervical cancer prevention services is available to all are warranted.
